# A novel mutation in *GRK1* causes Oguchi disease in a consanguineous Pakistani family

**Published:** 2009-09-05

**Authors:** Maleeha Azam, Rob W.J. Collin, Muhammad Imran Khan, Syed Tahir Abbas Shah, Nadeem Qureshi, Muhammad Ajmal, Anneke I. den Hollander, Raheel Qamar, Frans P.M. Cremers

**Affiliations:** 1 Department of Biosciences, COMSATS Institute of Information Technology, Islamabad, Pakistan; 2 Department of Human Genetics, Radboud University Nijmegen Medical Centre, Nijmegen, The Netherlands; 3 Department of Ophthalmology, Radboud University Nijmegen Medical Centre, Nijmegen, The Netherlands; 4 Nijmegen Centre for Molecular Life Sciences, Radboud University Nijmegen, Nijmegen, The Netherlands; 5 Vitreoretina Services, Al-Shifa Trust Eye Hospital, Rawalpindi, Pakistan; 6 Shifa College of Medicine, Islamabad, Pakistan

## Abstract

**Purpose:**

The purpose of this study was to identify the underlying molecular genetic defect in a large consanguineous Pakistani family with Oguchi disease who had been given a diagnosis of autosomal recessive retinitis pigmentosa.

**Methods:**

The family was genotyped with the Affymetrix 10K single nucleotide polymorphism array. Fine-mapping of a common homozygous region on chromosome 13q was performed using fluorescent microsatellite markers. Mutation analysis was done by direct sequencing of the candidate gene *GRK1* located in the region. The segregation of a novel mutation in the family and the frequency of the identified mutation in the Pakistani population were determined by StuI RFLP analysis.

**Results:**

Genetic mapping supported the diagnosis of typical Oguchi disease in a Pakistani family and also resulted in the identification of a novel nonsense mutation (c.614C>A; p.S205X) in exon 1 of *GRK1*. This mutation is predicted to result in premature termination of the protein product, thereby affecting the phototransduction cascade. A clinical reappraisal of the family revealed that all patients homozygous for this variant had Oguchi disease.

**Conclusions:**

This is the first report to describe a mutation causing typical Oguchi disease in a large consanguineous Pakistani family. This mutation segregated in eight affected members.

## Introduction

Oguchi disease is a rare autosomal recessive form of congenital stationary night blindness associated with fundus discoloration and abnormally slow dark adaptation after light exposure, along with characteristic electroretinographic (ERG) abnormalities. The disease was first reported by Oguchi in 1907 as a variant form of congenital stationary night blindness (CSNB) and was later characterized phenotypically by Mizuo in 1913 [1], who demonstrated the Mizuo-Nakamura phenomenon in affected individuals. In this test after 2–3 h of dark adaptation of the eyes, the diffused yellow or grey discoloration of the fundus returns to normal, along with the reappearance of rod function. The discoloration of the fundus reappears shortly after reexposure to light [2,3]. Oguchi disease has been shown to be more common in the Japanese population compared to other populations [4]. In addition to the typical Oguchi disease, variant forms have also been reported without the typical Mizuo-Nakamura phenomenon and variable fundus appearances and ERG patterns [5].

The first Oguchi locus was located on chromosome 2q37.1 by Maw et al. [6]. Subsequently, mutations in the arrestin, or S-antigen, gene (*SAG*) were found to be associated with Oguchi disease. Later, a mutation in a G-protein-dependent receptor kinase 1 (*GRK1*), also called rhodopsin kinase, which is located on chromosome 13q34, was shown to result in the Oguchi phenotype [7]. The proteins encoded by these two genes are members of the phototransduction pathway in rod photoreceptor cells [4,7]. Here, rhodopsin kinase (RK) and SAG work sequentially in deactivating the photoactivated rhodopsin, thereby stopping the phototransduction cascade.

Rhodopsin kinase is a rod-specific cytosolic enzyme, and its kinase activity is specifically directed to photoactivated rhodopsin by phosphorylating multiple serine residues [8]. Various types of mutations, e.g. both missense and protein truncating, have been shown to result in a decrease in the catalytic activity of the protein, leading to delayed photoreceptor recovery. These mutations are mainly associated with Oguchi disease [7,9,10], although one report describes the association of *GRK1* mutations with retinitis pigmentosa (RP) [11].

In this paper, we report a novel nonsense mutation in *GRK1* in affected members of a large consanguineous Pakistani family. These patients initially were diagnosed with RP. After identifying the genetic defect in affected family members, they were clinically re-evaluated through dark adaptation testing. The affected individuals were diagnosed with typical Oguchi disease, a phenotype that has thus far not been described in the Pakistani population.

## Methods

### Clinical evaluations

**Figure 1:**
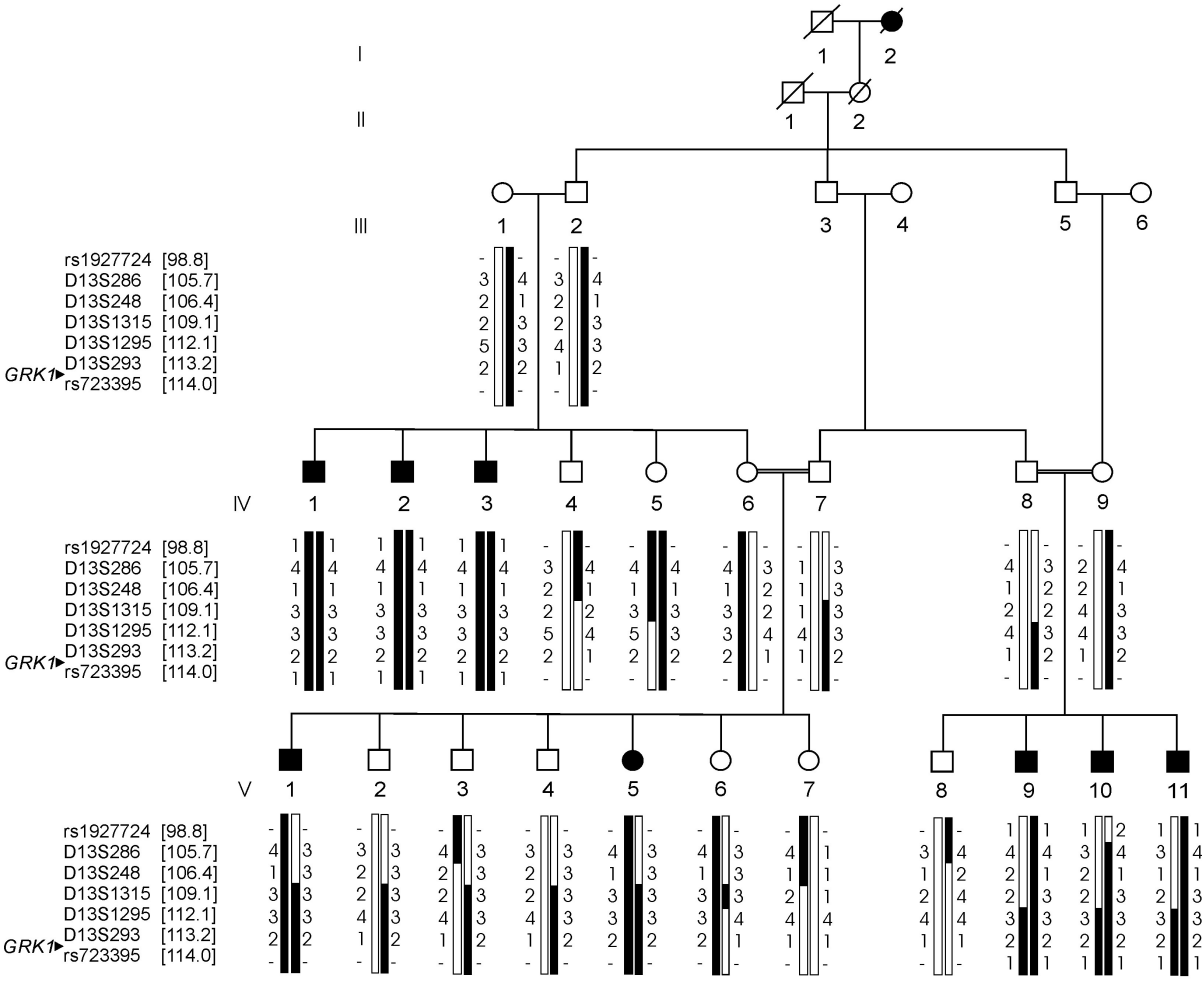
RP19 pedigree and 13q32.3-qter haplotypes. White circles represent healthy females, filled circles represent affected females, white squares represent healthy males, and filled squares are affected males. Slashed symbols indicate deceased individuals. Arrowhead marks the position of the *GRK1* gene at 13q34. The genomic positions (in Mb) of all the markers are based on UCSC genome working draft hg18 (March 2006) and indicated between brackets.

Twenty-two individuals from family RP19, including seven females and 15 males ranging in age from three to 66 years, were ascertained from the central region of Punjab province of Pakistan. The eight affected members had previously been diagnosed by a local doctor with autosomal recessive RP based on their night blindness. Ninety-three healthy individuals (26 females, 67 males, age 13–60 years) some from central Punjab and most from different regions of Pakistan, were interviewed to exclude the presence of RP or any other eye disease. Blood samples were collected from 22 individuals (eight affected and 14 healthy individuals) from a five-generation pedigree (Figure 1) who all consented to participate in the study. In two branches (IV6/IV7, IV8/IV9, and their children) the parents are first cousins. A consanguineous relationship between III-1 and III-2 is not known, although they belong to the same community. The medical and family history of the participants was then taken through a questionnaire. Fundoscopy without dark adaptation test and ERG were performed that didn’t give clear characteristics of Oguchi disease. Fundoscopy and electroretinographic (ERG) diagnostic tests were performed for selected members (IV-3, V-1, and V-2) of the RP19 family. This study was approved by the Shifa College of Medicine/Shifa International Hospital Ethics Committee/Institutional Review Board.

Before the ERG measurements were made, photoreceptor cells were first dark adapted (scotopic ERG) for 20–30 min to measure the rod response. Next, the patients were light adapted for 10 min (photopic ERG) to measure the cone response. The total (or aggregate) electrical response of the retina was then recorded with a contact lens electrode using commercial ERG equipment (LKC Inc., Gaithersburg, MD) [12].

To confirm the diagnosis of Oguchi disease in this family, individuals IV-3, V-1, and V-2 were studied by a dark adaptation test of 2–3 h to observe the changes in the fundus before and after the onset of light exposure. The fundi were examined by an indirect ophthalmoscope and 20 diopter lens. The light intensity of the indirect ophthalmoscope was kept at the lower levels to gain more time to observe the changes in the retina.

### Molecular genetic studies

Blood samples were drawn using vacutainer tubes containing acid citrate dextrose (Becton Dickinson product no 364606, Franklin Lakes, NJ). DNA isolation was performed by Sambrook’s method (organic method) of DNA extraction using 4 ml of blood, as described previously [13]. Six affected members from the fourth and fifth generation (IV-1, IV-2, IV-3, V-9, V-10, V-11) of the family (Figure 1) were genotyped with the Affymetrix 10K single nucleotide polymorphism (SNP) array containing 10,204 SNPs (Affymetrix, Santa Clara, CA). Multipoint parametric linkage analysis and LOD score calculations were performed with the GeneHunter program in the EasyLinkage software package version 5.02 [14] using the Decode Genetics SNP map and the Asian allele frequencies. An autosomal recessive mode of inheritance with full penetrance was assumed, and the disease allele frequency was estimated at 0.001. Fine-mapping of the linkage interval on chromosome 13q was performed with fluorescently labeled microsatellite markers. These markers were amplified by the polymerase chain reaction (PCR) under standard PCR conditions, and the haplotypes were constructed based upon the allele sizes of the microsatellites. Positions of the microsatellite markers were derived from the Marshfield map. Two-point parametric linkage analysis and LOD score calculations of the microsatellite markers were performed by using the SuperLink package version 1.6 in the EasyLinkage software package.

Sequencing primers for *GRK1,* a candidate gene in the region, were as previously reported by Zhang et al. [5]. These were used to amplify all exons of *GRK1* in one of the affected individuals from each branch of the family, along with a control sample. The amplified PCR products were separated on agarose gel and purified with the Nucleospin DNA extraction kit (Nucleospin Extract II, Macherey-Nagel GmbH & Co, Duren, Germany). Purified products were directly sequenced using the corresponding primers and dye-termination chemistry (BigDye Terminator, version 3 on a 3730 or 2100 DNA analyzer; Applied Biosystems, Foster City, CA).

To determine the presence of the mutation identified in *GRK1*, we amplified exon 1 of this gene in all the family members of family RP19, 42 unrelated Pakistani probands with RP, and a panel of 93 Pakistani control individuals. The 552 bp fragment was then digested with the restriction enzyme StuI, and resolved on an agarose (1.5%) gel. PCR products with the *GRK1* mutation were digested into fragments of 186 and 366 bp.

## Results

**Figure 2:**
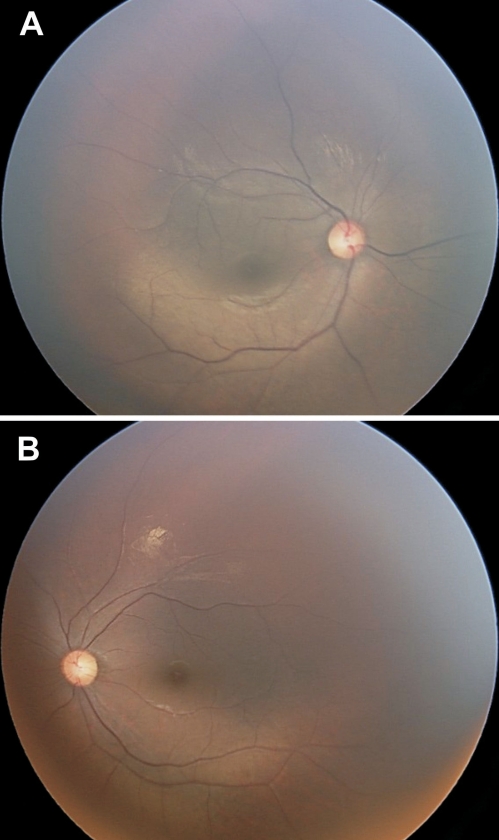
Fundus photographs of patient V-1 who has Oguchi disease. **A**: Fundus photograph of individual V-1 with Oguchi disease before dark adaptation. The gray discoloration of the fundus is a characteristic sign of Oguchi disease. **B**: Fundus photograph after 2 h of dark adaptation shows normal appearance of the retina.

Family RP19 was initially given a diagnosis of autosomal recessive RP. However, during fundoscopy of the individuals IV-3 and V-1, no bone spicule deposits were observed in the fundus (Figure 2A), which are typical for RP. Scotopic ERG showed a significantly reduced rod response (data not shown).

Six affected family members were analyzed by whole-genome SNP analysis, which showed homozygosity for a 15.2 Mb region on chromosome 13q34 between SNPs rs1927724 and rs723395 with a maximum LOD score of 3.29. The segregation of the haplotype among all available family members was confirmed and expanded by the fluorescently labeled microsatellite markers between rs1927724 and rs723395. In this way, the critical region was refined to a 5-Mb region between D13S1315 (position 109.1 Mb) at the centromeric side and 13qter (position 114.1 Mb). All eight affected individuals were homozygous for this chromosomal interval (Figure 1). At the telomeric side, no recombinations were observed between the Oguchi phenotype and the most telomeric analyzed SNP, rs723395. At the centromeric side, a recombination between D13S1315 and D13S1295 was deduced between the at risk haplotypes in the unaffected individuals III-1 or III-2 and IV-5. In addition, affected individuals V-9 and V-10 show a paternal at risk haplotype between D13S1315 and 13qter. We observed a maximum two-point LOD score of 5.1 for marker D13S1295 at theta 0.

**Figure 3:**
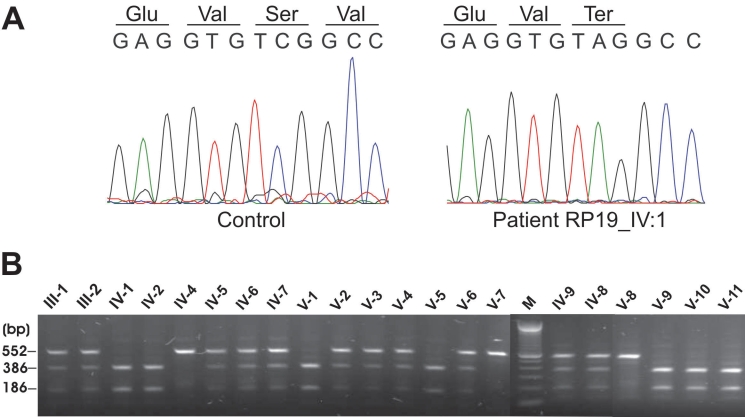
Sequence chromatograms and segregation analysis of the p.S205X *GRK1* variant. **A**: Sequence chromatogram of part of exon 1. Sequence of normal control sample (left panel) and an affected individual IV-1 (right panel) with the homozygous mutant sequence c.614C>A. **B**: StuI restriction analysis of the 552 bp PCR products of exon 1 in all family members. Fragments were resolved on an 1.5% agarose gel. Individuals with a single fragment of 552 bp were identified as homozygous normal since they did not contain the restriction site for StuI. Those with three fragments (552 bp, 366 bp, and 186 bp) were heterozygous carriers of the mutation, and those with two fragments (366 bp and 186 bp) carried the mutation homozygously.

One of the genes residing in the homozygous interval was *GRK1* (at 113.3 Mb), in which mutations have been reported [10] to cause Oguchi disease. All seven coding exons and flanking intronic sequences of *GRK1* were sequenced in affected family members IV-1, V-5, and V-11. Sequencing revealed a homozygous change of nucleotide C>A at position 614 in exon 1 of the *GRK1* gene in these three patients. This nucleotide change results in a nonsense mutation, p.S205X (Figure 3A).

The novel mutation c.614C>A results in the creation of a restriction site for the StuI restriction enzyme. To determine the segregation of the mutation, we performed StuI restriction analysis in all family members, which showed that all parents, as well as several unaffected siblings, were heterozygous carriers of the mutation. Eight unaffected individuals were found to be homozygous for the wild-type sequence (Figure 3B). As expected, all affected individuals were homozygous for the mutation. No carriers of the c.614C>A variant were detected after mutation screening of 42 RP probands and 93 Pakistani controls.

Because mutations in *GRK1* are mainly associated with Oguchi disease, clinical characteristics of the affected family members were reevaluated. Patients of family RP19 turned out to have an Oguchi phenotype, since the affected individuals have congenital stationary night blindness and grey discoloration of the fundus, which are characteristic signs of the disease. The typical Mizuo-Nakamura phenomenon of fundus changes for Oguchi was observed in the affected individuals who were subjected to a dark adaptation of only 2 h. The normal recommended time of dark adaptation is at least 2–3 h to see a clear difference between the light and dark adapted fundus. The Mizuo-Nakamura phenomenon in the participants of our study exhibited a less marked disappearance of the fundus' unusual gray discoloration and the appearance of the reddish coloration due to reduced time for dark adaptation (Figure 2B).

## Discussion

In this study, we present a Pakistani family (RP19) with Oguchi disease in whom we found a causative novel protein-truncating mutation in the *GRK1* gene (p.S205X). The affected members have congenital stationary night blindness along with diffuse gray discoloration of the fundus and show the Mizuo-Nakamura phenomenon after dark adaptation of 2 h. ERG data showed nonrecordable rod responses, which is characteristic for patients affected with Oguchi disease [15]. The Mizuo-Nikamura phenomenon is known to be a specific sign of Oguchi disease, an unusual form of congenital stationary night blindness, but has also been described in X-linked recessive and dominant cone dystrophy, Stargardt disease, and X-linked retinoschisis [16-19].

In the Japanese and European population, several cases have been reported in which the causative mutations are either in the *SAG* gene, or in the *GRK1* gene [20-22]. Oguchi disease is rare worldwide, while the prevalence is the highest in the Japanese population [23]. A variant form of Oguchi disease has also been reported to be caused by a deletion in exon 3 of the *GRK1* gene without the typical Mizuo-Nakamura phenomenon in the affected individual [5].

The *GRK1* gene encodes rhodopsin kinase, a cytosolic enzyme that belongs to the family of signal-transduction proteins and mediates cellular responses in the rod photoreceptor cells. It is a serine-threonine kinase, which plays a key role in normal deactivation and recovery of the photoreceptor after exposure to light [9]. In the retina, desensitization of rod cells occurs when light-activated rhodopsin is phosphorylated by rhodopsin kinase, followed by the binding of visual arrestin to the phosphorylated rhodopsin. In turn, this blocks the interaction of rhodopsin with transducin, which results in switching off the phototransduction cascade [24]. As such, rhodopsin kinase is a crucial factor in regulating the phototransduction cascade and any defect in its expression is predicted to affect the normal functioning of the retina.

The work of Choi et al. [25] on *Sag*/*Grk1*-deficient mouse retina has demonstrated the functional importance of rhodopsin kinase in the phototransduction process. They showed that when the animals are raised in the dark, they have normal retinal morphology. However, when exposed to continuous light, these animals show rapid induction of photoreceptor cell death that appears to be the result of apoptosis. Chen et al. [26] found that the retina of *Grk*^−/−^ mice raised in darkness had the same morphology as *Grk*^+/+^ mice. However, constant light exposure of *Grk*^−/−^ mice resulted in degeneration, causing light-induced apoptosis in the rods lacking Grk1.

The biochemical effect of the mutations in *GRK1* in patients with Oguchi disease has been studied by Khani et al. [9], who found the compound heterozygous p.V380D and p.S536fsX6 variants in a European patient. A comparison of the functional characteristics of the wild-type and these two mutant proteins showed that both mutant proteins had decreased catalytic activity, demonstrating their pathogenic nature [9]. In addition to the *GRK1* missense, null and frameshift mutations identified by different groups, many Oguchi disease patients have been shown to carry deletions of one or more exons of the gene [4-9]. The novel nonsense mutation (p.S205X) in *GRK1* in RP19 described here is located in the catalytic protein kinase domain (aa196–219), and is predicted to result in premature termination of the protein product and as such results in a nonfunctional rhodopsin kinase.

Our study has resulted in the identification of the first typical Oguchi family in Pakistan with a novel mutation in *GRK1*. This study also demonstrates the power of genetic studies to correctly diagnose the disease. Previously this family was classified as having RP, but our diagnostic clinical tests and genetic data revealed that this family has Oguchi disease.

## References

[1] Oguchi C Über einen Fall von eigenartiger Hemeralopie. Nippon Ganka Gakkai Zasshi 1913 11 123

[2] Mizuo G On a new discovery in the dark adaptation on Oguchi’s disease. Acta societatis ophthalmologicae Japonicae 1913 17 1148

[3] Usui T Ichibe M Ueki S Takagi M Hasegawa S Abe H Sekiya K Nakazawa M Mizuo phenomenon observed by scanning laser ophthalmoscopy in a patient with Oguchi disease. Am J Ophthalmol 2000 130 359 61 11020420 10.1016/s0002-9394(00)00532-8

[4] Fuchs S Nakazawa M Maw M Tamai M Oguchi Y Gal A A homozygous 1-base pair deletion in the arrestin gene is a frequent cause of Oguchi disease in Japanese. Nat Genet 1995 10 360 2 7670478 10.1038/ng0795-360

[5] Zhang Q Zulfiqar F Riazuddin SA Xiao X Yasmeen A Rogan PK Caruso R Sieving PA Riazuddin S Hejtmancik JF A variant form of Oguchi disease mapped to 13q34 associated with partial deletion of GRK1 gene. Mol Vis 2005 11 977 85 16319817

[6] Maw MA John S Jablonka S Muller B Kumaramanickavel G Oehlmann R Denton MJ Gal A Oguchi disease: suggestion of linkage to markers on chromosome 2q. J Med Genet 1995 32 396 8 7616550 10.1136/jmg.32.5.396 PMC1050438

[7] Hayashi T Gekka T Takeuchi T Goto-Omoto S Kitahara K A novel homozygous GRK1 mutation (P391H) in 2 siblings with Oguchi disease with markedly reduced cone responses. Ophthalmology 2007 114 134 41 17070587 10.1016/j.ophtha.2006.05.069

[8] Ohguro H Van Hooser JP Milam AH Palczewski K Rhodopsin phosphorylation and dephosphorylation in vivo. J Biol Chem 1995 270 14259 62 7782279 10.1074/jbc.270.24.14259

[9] Cideciyan AV Zhao X Nielsen L Khani SC Jacobson SG Palczewski K Null mutation in the rhodopsin kinase gene slows recovery kinetics of rod and cone phototransduction in man. Proc Natl Acad Sci USA 1998 95 328 33 9419375 10.1073/pnas.95.1.328 PMC18214

[10] Khani SC Nielsen L Vogt TM Biochemical evidence for pathogenicity of rhodopsin kinase mutations correlated with the Oguchi form of congenital stationary night blindness. Proc Natl Acad Sci USA 1998 95 2824 7 9501174 10.1073/pnas.95.6.2824 PMC19653

[11] Yamamoto S Khani SC Berson EL Dryja TP Evaluation of the rhodopsin kinase gene in patients with retinitis pigmentosa. Exp Eye Res 1997 65 249 53 9268593 10.1006/exer.1997.9998

[12] Standard for clinical electroretinography. International Standardization Committee. Arch Ophthalmol 1989 107 816 9 2730397 10.1001/archopht.1989.01070010838024

[13] Sambrook J, Fritsch EF, Maniatis T. 1989. Molecular Cloning: A Laboratory Manual (2nd ed.), Cold Spring Harbour Laboratory Press, Cold Spring Harbor, New York.

[14] Lindner TH Hoffmann K easyLINKAGE: A PERL script for easy and automated two-/multi-point linkage analyses. Bioinformatics 2005 21 405 7 15347576 10.1093/bioinformatics/bti009

[15] Yamamoto S Hayashi M Takeuchi S Shirao Y Kita K Kawasaki K Normal S cone electroretinogram b-wave in Oguchi’s disease. Br J Ophthalmol 1997 81 1043 5 9497461 10.1136/bjo.81.12.1043 PMC1722076

[16] Heckenlively JR Weleber RG X-linked recessive cone dystrophy with tapetal-like sheen. Arch Ophthalmol 1986 104 1322 8 3489456 10.1001/archopht.1986.01050210076029

[17] Pinckers A Deutman AF X-linked cone dystrophy. An overlooked diagnosis. Int Ophthalmol 1987 10 241 3 3498700 10.1007/BF00155631

[18] Noble KG Margolis S Carr R The golden tapetal sheen reflex in retinal disease. Am J Ophthalmol 1989 107 211 7 2923149 10.1016/0002-9394(89)90302-4

[19] de Jong PTVM Zrenner E van Meel GJ Keunen JEE van Norren D Mizuo Phenomenon in X-linked Retinoschisis. Pathogenesis of the Mizuo phenomenon. Arch Ophthalmol 1991 109 1104 8 1867553 10.1001/archopht.1991.01080080064029

[20] Yamamoto S Sippel KC Berson EL Dryja TP Defects in the rhodopsin kinase gene in the Oguchi form of stationary night blindness. Nat Genet 1997 15 175 8 9020843 10.1038/ng0297-175

[21] Yoshida S Yamaji Y Yoshida A Ikeda Y Yamamoto K Ishibashi T Rapid detection of SAG 926delA mutation using real-time polymerase chain reaction. Mol Vis 2006 12 1552 7 17200654

[22] Oishi A Akimoto M Kawagoe N Mandai M Takahashi M Yoshimura N Novel mutations in the GRK1 gene in Japanese patients with Oguchi disease. Am J Ophthalmol 2007 144 475 7 17765441 10.1016/j.ajo.2007.03.025

[23] Carr RE Gouras P Oguchi's disease. Arch Ophthalmol 1965 73 646 56 14281981 10.1001/archopht.1965.00970030648010

[24] Horner TJ Osawa S Schaller MD Weiss ER Phosphorylation of GRK1 and GRK7 by cAMP-dependent protein kinase attenuates their enzymatic activities. J Biol Chem 2005 280 28241 50 15946941 10.1074/jbc.M505117200

[25] Choi S Hao W Chen CK Simon MI Gene expression profiles of light-induced apoptosis in arrestin/ rhodopsin kinase-deficient mouse retinas. Proc Natl Acad Sci USA 2001 98 13096 101 11687607 10.1073/pnas.201417498 PMC60830

[26] Chen CK Burns ME Spencer M Niemi AG Chen J Hurley J Baylor DA Simon MI Abnormal photoresponses and light-induced apoptosis in rods lacking rhodopsin kinase. Proc Natl Acad Sci USA 1999 96 3718 22 10097103 10.1073/pnas.96.7.3718 PMC22360

